# Biomonitoring for Improving Alcohol Consumption Surveys: The New Gold Standard?

**DOI:** 10.35946/arcr.v36.1.05

**Published:** 2014

**Authors:** Thomas K. Greenfield, Jason Bond, William C. Kerr

**Affiliations:** Thomas K. Greenfield, Ph.D., is center director and scientific director; Jason Bond, Ph.D., is senior statistician; and William C. Kerr, Ph.D., is senior scientist at the Alcohol Research Group, Public Health Institute, Emeryville, California.

**Keywords:** Alcohol consumption, alcohol consumption pattern, alcohol consumption measurement, alcohol consumption surveys, monitoring alcohol levels, measurement tools, biological tools, assessment, biomonitor, WrisTAS^™^, SCRAM^™^

## Abstract

To assess alcohol consumption levels in large populations, researchers often rely on self-report measures. However, these approaches are associated with several limitations, particularly underreporting. Use of noninvasive biomonitoring approaches may help validate self-report alcohol consumption measurements and thus improve their accuracy. Two such devices currently are available, the WrisTAS^™^ and SCRAM^™^ devices, both of which measure alcohol vapors emitted through the skin after alcohol consumption. Several studies assessing the utility of the WrisTAS^™^ bracelet in determining alcohol consumption levels noted that it was associated with relatively high failure rates. The SCRAM^™^ is an ankle bracelet intended for court-ordered alcohol monitoring. In studies, its sensitivity exceeded that of the WrisTAS^™^ and increased with increasing blood alcohol concentrations. Although early studies also identified some equipment concerns with the SCRAM^™^, studies of its ability to detect moderate and heavy drinking recently have yielded good results. Biomonitoring devices already are valuable tools and with further improvements may become even more useful in both research and practical applications**.**

An important focus of alcohol research is measuring alcohol consumption levels of larger populations to get a better understanding of real-life consumption patterns as well as the health and social consequences of drinking. Although this sounds fairly straightforward, it actually is quite challenging to devise measurement tools that allow for accurate and reliable reporting of alcohol consumption by larger numbers of subjects in surveys, with a low reporting burden to the participants. Measurements of blood alcohol concentrations (BACs) would provide the most reliable information on a key biological counterpart to alcohol consumption but are invasive and thus not feasible outside of very limited, usually laboratory-based studies. Breath alcohol concentration (BrAC) measurements are less invasive to obtain, but to be useful they also require demanding, repeated measurements. Self-reports of alcohol consumption (e.g., via drinking diaries or summary measures) are more convenient and can be implemented even in studies that include large numbers of participants; however, these self-reports also have their drawbacks, principally potential recall bias. Consequently, researchers are looking at developing other tools for monitoring alcohol levels through objective biological measures in a convenient, noninvasive manner.

This article explores how biological measures have been used to validate and increase the accuracy of self-reports of alcohol consumption in general-population epidemiologic surveys. After reviewing some of the limitations and difficulties associated with self-report alcohol consumption measures, the article briefly reviews studies involving two biomonitoring devices—the SCRAM™ device and the WrisTAS™ device—and describes in more detail a study that compared alcohol consumption reported via traditional self-report drinking diaries and period summaries with measurements using the WrisTAS™ device. It is important to note, however, that these findings to date are at an early stage and additional, well-controlled studies are necessary to confirm the findings. The article also briefly touches on the benefits and limitations associated with such biomonitoring devices as well as their value for improving alcohol intake pattern measurement in general population surveys.

## Self-Report Alcohol Measurements and Their Limitations

The most commonly used approach to determining people’s drinking levels in various studies is self-reported alcohol consumption as established using such assessment tools as quantity–frequency, graduated-frequency (GF), short-term recall, or time-line follow-back (TLFB) measures. Quantity–frequency measures simply ask respondents how often they drink and what amount of alcohol (i.e., how many drinks) they typically consume on each day or each drinking occasion. These measures can be expanded by distinguishing between beverage types, adding questions on binge or episodic heavy drinking, and assessing drinking patterns over different recall periods (Rehm 1998). GF measures attempt to get a more accurate measure of actual alcohol volumes consumed by grouping the number of drinks consumed in a day into graduated categories. Starting with the maximum number of drinks (Greenfield et al. 2006) respondents report having consumed on 1 day during a specified time period (e.g., the past month or year), they are asked how often they have drunk progressively fewer drinks per day during the same time period. This approach allows researchers a better estimate of specific drinking patterns and improves the accuracy of the estimate of total consumption (Greenfield 2000). Short-term recall measures ask respondents to recall all alcohol that they have consumed during each day in a recent short period (e.g., the last week) (Rehm et al. 1999), based on the assumption that respondents are able to remember consumption more accurately over such short periods. A similar approach is the TLFB method (Sobell and Sobell 2000), which asks respondents to provide retrospective estimates of their drinking using a calendar, going back in time from the time of the interview. Additional memory aids can be used to enhance the respondents’ recall, such as key dates that serve as anchors for reporting drinking. This method is recommended when relatively precise estimates of drinking are needed, but is rarely used in large-scale research.

Self-report alcohol consumption measurement using well-developed measures from these approaches under appropriate assurances of confidentiality and anonymity has been considered reasonably accurate for research purposes (Del Boca and Darkes 2003; Midanik 1988). Nevertheless, it does have several limitations. For example, the consumption levels reported by existing national surveys typically account for only a relatively small proportion of the overall per capita consumption derived from alcohol sales data. This deviation gives rise to a general concern regarding underreporting in surveys relying on self-reported consumption (Greenfield and Kerr 2008). Also, researchers do not know whether the tendency to underreport drinking levels is similar across all drinkers or is greater among heavier drinkers (Feunekes et al. 1999). Several ways to correct this situation by refining or adding to these survey self-reports have been suggested, including the following:

- Measuring quantity and frequency of drinking across a range of drinking contexts (Casswell et al. 2002);
- Using detailed “yesterday” measures together with more standard self-report measures (Stockwell et al. 2008) or recent recall and/or prospective drinking-diary methods for calibration and correction of more standard pattern measures, such as the GF measure (Greenfield et al. 2009*b*, 2010; Hilton 1989); and
- Carefully collecting information on the drinker’s typical or recent drink strength and size and applying this as a correction factor to the number of “drinks” reported (Greenfield and Kerr 2008; Greenfield et al. 2010; Kerr and Greenfield 2007). This approach is based on the observation that most “drinks” typically consumed in real life exceed the pure ethanol amount of 0.6 oz (14 g) that is contained in a U.S. standard drink, particularly for wine and spirits. This is true for both drinks consumed at home (Kerr et al. 2005) and those consumed in on-premise drinking venues (Kerr et al. 2008, 2009*b*) and may be especially pronounced in certain ethnic minority groups (Kerr et al. 2005, 2009*a*). One estimate placed the average alcohol content consumed with typical drinks as 32 percent above the alcohol content of a standard drink (Greenfield et al. 2009*b*).

Despite these strategies, self-report measures of alcohol consumption will always be associated with some degree of subjectivity, and therefore objective measures that can easily, accurately, and reliably monitor alcohol consumption are desirable. Importantly, they offer the promise of helping validate survey self-reports.

## Biomonitors to Improve Accuracy of Alcohol Consumption Measurements

The validation of survey self-reports against either retrospective or prospective diaries has been called validation against a “silver” standard (Greenfield et al. 2009*b*). Attempts to validate against a physiological measure have been called validation against a “gold standard” (Swift et al. 2010). There currently are two noninvasive alcohol detection devices that have been used in gold-standard validations, the SCRAM™ and the WrisTAS™. Both devices indirectly measure BACs, and thus, by inference, alcohol consumption, by assessing alcohol vapors that are eliminated through the skin in perspiration. In general, approximately 1 percent of ethanol ingested is eliminated from the human body this way. The SCRAM™ measures this transdermal alcohol concentration (TAC) using fuel-cell technology, whereas the WrisTAS™ uses an oxidation current.

### The SCRAM^™^ and Its Use in Determining AlcoholConsumption

The Secure Continuous Remote Alcohol Monitoring (SCRAM™) device for measuring TAC, which was designed by Alcohol Monitoring Systems, Inc., measures the alcohol vapors above a small surface area enclosed by a rubber muff. The device is worn around the ankle and is locked, so that it cannot be removed by the wearer. It may be worn around the clock in most everyday situations (e.g., even in the shower, although it cannot be immersed in water). The SCRAM™ takes measurements every 15, 30, or 60 minutes (Sakai et al. 2006), 30 minutes being the typical interval (Barnett et al. 2014). The device is available commercially to law-enforcement agencies and was designed for people undergoing court-ordered alcohol monitoring, such as those convicted of driving while intoxicated. Either TAC readings above a specified level (typically those corresponding to a BAC of 0.02 g/dL) or attempts to remove or defeat the device result in a report sent through a central server, which receives data transmitted daily (Marques and McKnight 2007, 2009).

In an early study conducted by the National Highway and Traffic Safety Administration (NHTSA) that evaluated the accuracy and reliability of the SCRAM™ device as well as the WrisTAS™, the SCRAM™ failed to respond to alcohol consumption in 15 percent of the drinking episodes (Marques and McKnight 2007, 2009). However the sensitivity of the device increased with increasing BACs. Thus, the device accurately detected 57 percent of the drinking episodes where the BAC was 0.02 to 0.08 g/dL, but 88 percent of the episodes where the BAC exceeded 0.08 g/dL.

More recent research experience shows improved performance of the SCRAM™ (Barnett et al. 2011). In a study reanalyzing data from earlier contingency management studies involving heavy-drinking adults, Barnett and colleagues (2014) examined the ability of the SCRAM™ to detect drinking episodes recorded by daily Web-based self-reports. The reports included the number of standard drinks consumed and times of beginning and ending drinking. Estimated BACs (eBACs) could be calculated from these data. Including instances when the device was functional (with minimal malfunctions noted), the researchers analyzed 690 drinking episodes, with a mean of 8 episodes per participant and a mean of 6.3 drinks per episode (standard deviation of 4.5). The findings indicated that the sensor detected 72.8 percent of self-reported drinking episodes. Detection rates differed by gender, with the sensor detecting 77 percent of episodes in women and 69 percent of episodes in men; this difference was not statistically significant. However, further analyses assessing an interaction between gender and heavy drinking found that at drinking levels of less than five drinks per episode, the SCRAM™ was more likely to detect drinking in women (53.4 percent) than in men (32.6 percent), whereas no gender difference was observed at drinking levels of five or more drinks (women 92.6 percent; men 93.4 percent). The investigators suggested that at lower drinking levels, women’s relatively higher eBACs led to higher TACs, thereby contributing to better detection, as also seen for both genders in the heavy-drinking episodes. The weighted correlation between TAC and eBAC was reported to be 0.54 (*p* < 0.001). In turn, eBAC was highly correlated with self-reported number of drinks in the episode (*r* = 0.77, *p* < 0.001). Although body mass index, current alcohol dependence, and number of drinks were each associated with detection in univariate analyses, in a multivariate GEE analysis, however, only number of drinks was significantly associated with TAC detection. The investigators concluded that these findings indicate the utility of the SCRAM™ for general use because its performance only was influenced by level of alcohol consumption but not by user characteristics.

Other studies have assessed the correlation of TAC readings with number drinks consumed over a certain period (e.g., 8 days) (Sakai et al. 2006), or during one event (Barnett et al. 2014). These studies have provided evidence that the quantity in standard drinks also can be assessed from SCRAM™derived TAC readings. Although individual differences— for example, in metabolism rate (Edenberg 2007)—make the individual relationship noisy, on a group basis the mean relationships between number of drinks and TAC were strong (Barnett et al. 2014). In one recent laboratory study (Hill-Kapturczak et al. 2014), 11 men and 10 women were administered increasing doses of beer (from one to five drinks). Each participant consumed each ascending quantity on successive days, and all drinks were consumed at set intervals over a 2-hour period. The researchers investigated differences between mean BrAC and mean SCRAM™-based TAC time courses for men and women. For three men and five women (38 percent of participants), the SCRAM™ detected no TAC after consumption of one beer; for the remaining participants, as well as for all participants when they consumed two or more beers, however, the SCRAM™ showed a non-zero TAC. The investigators first assessed the influence of sex and consumption level on BrAC and TAC curves. For the mean BrAC curves by gender and time, strong “sex differences were an increasing function of beers consumed” (Hill-Kapturczak 2014, p. 5)—that is, sex differences increased the more beer the participants had consumed. For TAC mean curves by gender and time, in contrast, there were highly significant effects of beers consumed but only modest nonsignificant sex differences were seen (as a main effect trend, *p* < 0.1). A critical finding was that time to peak for both BrAC and TAC was a clear function of the number of drinks. Interestingly, the time lag between BrAC and TAC, which averaged just over 2 hours (129 minutes), in reality also was a function of number of beers consumed (*p* < 0.001). Finally, the researchers developed a parsimonious equation based on the model predicting peak BrAC from TAC data, taking into account the peak TAC, time-to-peak TAC, sex, and a peak TAC–by–sex interaction. This equation estimate was highly correlated with peak BrAC, accounting for 76 percent of the variance; it also was validated using a previous dataset (Dougherty et al. 2012) that had demonstrated a very high correlation (Spearman *r* = 0.86, *p* < 0.0001) between peak BrAC and its equation-derived estimate from TAC records. Taken together, the results of the studies by Hill-Kapturczak and colleagues (2014) and Dougherty and colleagues (2012) suggest that it is possible to estimate peak BrACs from TAC readings when accounting for possible sex-related differences.

In summary, the results of these laboratory studies using transdermal monitors such as the SCRAM™ for validating drinking-pattern self-reports indicate that TAC readings are related to peak BrAC, which itself is strongly related to number of drinks. Accordingly, this approach and the derived equations could be used to estimate alcohol consumption (in the two- to five-drink range) from the TAC levels recorded by transdermal sensors. Other researchers have developed more complex distributed-parameter models reflecting transport of ethanol from blood through the skin into the transdermal sensor, to “deconvolute” TAC (in this case determined using WrisTAS™ data) into BAC, BrAC, and ultimately the number of drinks consumed in a given time period (Dumett et al. 2008; Rosen et al. 2014). Further work on such projects currently is continuing. Nevertheless, the existing data already indicate that TAC measurements are reasonably valid for research purposes, allowing investigators to use this approach, which may be more convenient and less burdensome to users, to assess alcohol consumption in surveys rather than detailed self-report measures.

### The WrisTAS^™^ and Its Use in Determining AlcoholConsumption

The noninvasive wrist-worn WrisTAS™ transdermal sensor (Swift 1993, 2000), developed by Giner, Inc., of Newton, Massachusetts, uses a patented electrochemical sensor to monitor patterns of drinking objectively, conveniently, and passively (Swift et al. 1992). The sensor measures the continuous electrochemical oxidation of ethanol vapor to acetic acid in a small space above the skin enclosed by a foam seal. Measurements can be taken every minute, or integrated into longer intervals (2 minutes, 5 minutes, etc.). The device also monitors skin temperature and conductivity to confirm that the person is indeed wearing the device (for an example of the readouts, see the figure). The same technology recently also has been incorporated into an ankle-worn alcohol monitor (BI-TAD^®^) for court-ordered remote alcohol monitoring.

Following the seminal work by Swift and colleagues (Swift 2000; Swift and Swette 1992; Swift et al. 1992), several recent studies have assessed the utility of the WrisTAS™ in monitoring alcohol consumption, with a focus on the relationship between TAC and self-reported drinking. Several of these projects have been funded by the National Institute on Alcohol Abuse and Alcoholism (NIAAA), including laboratory and 7-day field tests (Swift et al. 2010) and a study designed to improve self-report alcohol measures using 28-day drinking diaries and the WrisTAS™ (Bond et al. 2009*b*, 2014; Greenfield et al. 2009*a*). In addition, the previously mentioned study conducted by NHTSA (Marques and McKnight 2007, 2009) included the WrisTAS™, as did a study conducted by Wray and colleagues (2012).

Wray and colleagues (2012) compared alcohol measurements obtained with the WrisTAS™ with those obtained with three self-report measures (i.e., TLFB, random assessments during the day, and self-initiated daily morning assessments of previous-day drinking). Their analyses found that the WrisTAS™ correctly classified 86.1 percent of self-reported drinking events and showed high sensitivity (73.83) and specificity (92.44). Moreover, all three self-report measures were correlated with the transdermal readings; in general, each 0.01 g/dL increase in WrisTAS™-determined TAC peak corresponded to an increase in the number of reported drinks by 2.0 to 2.4 percent.

In the NIAAA-sponsored project on self-report alcohol measurement improvement (Bond et al. 2009*b*, 2014; Greenfield et al. 2009*a*), a prototype of the WrisTAS™ was used to validate self-report alcohol measures.^1^ In the experiment, which measured alcohol consumption over a 28-day period, participants were instructed to drink normally and assigned to one of three groups:

- Group 1, the reactivity control group, participated in no activities other than pre-and poststudy telephone interviews.
- Group 2, in addition to the interviews, completed daily paper-and-pencil drinking-event reports (diaries), including number of drinks consumed, brand and drink size, time and place of drinking occasions, with three different beverages (i.e., beer, wine, and spirits). This information was used to assess number of drinks and adjusted ethanol intake per drink and per day. The reports also identified exercise periods, eating food when drinking, and self-reported sweating (Swift and Swette 1992).
- Group 3, in addition to pre-and postsurveys and detailed diaries, wore the WrisTAS™ continuously for 2 consecutive weeks (i.e., either the first 2 or the last 2 weeks of the 4-week period)^2^ to monitor ethanol levels at 5-minute intervals.

For all three groups, their alcohol intake pattern over the preceding 28-day period was determined at the beginning and end of the 4-week study period through a telephone-administered interview. This interview included a standard 12-month GF measure (Greenfield 2000), as well as a newly developed, 28-day GF measure that asked for the maximum number of drinks a participant had consumed in the prior 28 days and the number of days that he or she drank at that and successively lower levels.

Pilot data determined that compliance was very high, with 94 percent of participants completing all study participation objectives. However, the device had a relatively high failure rate because it did not function properly 43 percent of the time. Of the interpretable person-days, the WrisTAS™ measurements and diaries agreed on 76 percent of person-days. Finally, the peak measurements recorded by the WrisTAS™ seemed to correspond with the alcohol quantities reported in respondents’ diaries (Greenfield et al. 2005).

During the experimental phase, no significant differences were detected in 28-day alcohol consumption reported at the beginning and end of the study for any of the three groups (Bond and Greenfield 2007). The changes in the number of drinking days (i.e., drinking frequency) were negligible for all three groups. Thus, the overall impact of the experimental manipulations (i.e., 14 days of TAS monitoring and/or daily drinking diaries) on participants’ natural drinking behavior was minimal, suggesting low reactivity to either of the intensive measurement protocols (Groups 2 and 3) compared with pre-and postassessments only (Group 1). Other studies, however, have suggested that weak reactivity to continuous biomonitoring may occur. For example, Neville and colleagues (2013) compared alcohol consumption in undergraduate students who were instructed not to drink over a 2-week period with those who were instructed not to drink and also wore a SCRAM™ device. Compared with a control group who also wore the SCRAM™ but had not been asked to stop drinking, both groups who had been instructed not to drink significantly reduced their consumption. Furthermore, significantly more participants wearing the SCRAM™ were able to abstain compared with those not wearing the device, although this difference disappeared when an intent-to-treat analysis of the data was utilized. Nonetheless, the findings suggest that use of the device itself may affect drinking behavior.

### Limitations of Biomonitoring Devices

Despite their potential usefulness in accurately measuring alcohol consumption, the currently available transdermal monitoring devices still suffer from some limitations. First, they are rather costly, limiting their use in larger studies. Second, although not required, it often is useful for each person wearing such a device to have it calibrated before wearing it out in the field—for example, by consuming a standard alcohol dose (e.g., two standard drinks) and then measuring the resulting TAC (Swift et al. 2010). This is particularly true for the WrisTAS™, where such a step also helps ensure that the device is functioning well at the start. Newer versions of the SCRAM™ seem to have achieved improved reliability so that for research in field settings participants need only receive initial training in the use of the device without calibration before using it in their normal lives. The new deconvolution estimation techniques (Dumett et al. 2008) now being developed should further reduce the need for laboratory calibration steps, and even the simpler TAC-to-BrAC estimation strategies (Hill-Kapturczak et al. 2014) seem to be effective for experimentally grouped data.

Second, the quality of the biomonitoring devices and their TAC readouts requires further improvement to achieve their full potential. For example, in the WrisTAS™ self-report measurement improvement study (Greenfield et al. 2005), equipment malfunction occurred approximately 50 percent of the time. Although this seemed to be largely a random process that did not skew the results, further improvements in functionality are warranted, and are being developed by the manufacturers. Equally important is increased convenience of analyzing the devices’ TAC output so as to extract BACs, BrACs, and estimates of the number of drinks consumed in particular periods.

## Conclusions

Biomonitors such as the WrisTAS™ and SCRAM™ have numerous potential uses, both in research settings and in practical applications. In research projects they can allow investigators to capture estimates of BACs resulting from naturalistic drinking events. This type of data could be useful particularly in epidemiological research because, as described here, traditional self-report measures may underestimate the respondents’ actual alcohol intake, particularly when no information on drink sizes and strengths consumed is available and standard drink sizes and alcohol content are assumed. Thus, studies found that the alcohol content of a typical drink may exceed that of an assumed standard drink by as much as 32 percent (Greenfield et al. 2009*b*; also see Bond et al. 2014). Because of these potential discrepancies, both epidemiological and treatment studies could benefit from the use of a gold standard, such as biomonitoring using transdermal sensors, rather than self-report measures, such as recent-recall and diary data, which can only be considered a silver standard. Additionally, biomonitoring devices have more practical applications; thus, the SCRAM™ and the newer Giner-technology anklets already are being used in criminal-justice settings to monitor alcohol consumption in drivers convicted of driving while intoxicated.

Once the current limitations of biomonitoring devices have been resolved, use of these devices is likely to become more accepted for a variety of study designs. For example, they may provide useful new tools for both verifying self-report alcohol summaries and for many continuous alcohol-monitoring applications in real-life settings.

## Figures and Tables

**Figure:**
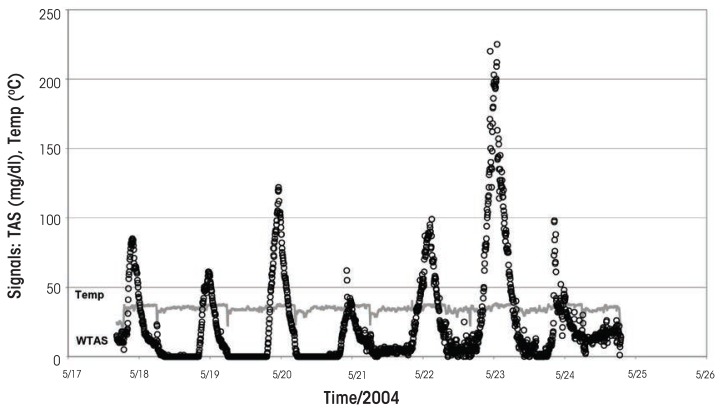
Example of a TAS readout showing the alcohol signal (dots) and temperature readings (shaded line).

## Footnotes

1 For the validity analyses reported here, the investigators used simple estimates of the alcohol amount consumed during a particular drinking event as indicated by the peak readings in mg ethanol per deciliter blood (mg/dL) and the area under the curve obtained from the recorded output (Bond et al. 2009*a*, 2014).

2 Participants received the WrisTAS™ version 5 during early stages of the study; this was upgraded to version 6 and finally to version 7 in the late phase, when the manufacturer obtained NIAAA-supported small business innovation research (SBIR) funding for the device’s further development. To minimize device data loss resulting from malfunction or miscalibration, the WrisTAS™ was replaced with a second device for week 2.
